# Personality Features in Obesity

**DOI:** 10.3389/fpsyg.2020.530425

**Published:** 2021-01-14

**Authors:** Livia Buratta, Chiara Pazzagli, Elisa Delvecchio, Giulia Cenci, Alessandro Germani, Claudia Mazzeschi

**Affiliations:** ^1^Department of Philosophy, Social Sciences, Humanities and Education, University of Perugia, Perugia, Italy; ^2^Healthy Lifestyle Institute, Centro Universitario Ricerca Interdipartimentale Attività Motoria (CURIAMO), University of Perugia, Perugia, Italy

**Keywords:** obesity, personality, assessment, psychopathology, borderline

## Abstract

Obesity is a widespread and broadly consequential health condition associated with numerous medical complications that could increase mortality rates. As personality concerned individual’s patterns of feeling, behavior, and thinking, it may help in understanding how people with obesity differ from people with normal-weight status in their typical weight-relevant behavior. So far, studies about personality and BMI associations have mainly focused on broad personality traits. The main purpose of this study was to explore the personality and health associations among a clinical group composed of 46 outpatients with overweight/obesity (mean age = 55.83; SD = 12.84) in comparison to a healthy control group that included 46 subjects (mean age = 54.96; SD = 12.60). Both the clinical and control groups were composed of 14 males and 32 females. Several personality and psychopathological aspects were assessed with the Personality Assessment Inventory (PAI). The results of the analysis of variance of aligned rank transformed (ART) showed that patients with overweight/obesity reported higher scores for Somatic Complaints, Depression, and Borderline Features than the control group. Logistic regression highlighted specifically that the subscales of the Borderline Features assessing the Negative Relationship contributed to the increased risk of belonging to the clinical group. For the purpose of this study, the role of gender was considered. The present findings highlight the importance of focusing on assessing personality functioning in the health context and on specific characteristics of interpersonal relationships to promote more tailored treatments.

## Introduction

The association between personality and health issues has been widely detected showing a co-occurrence between certain personality characteristics and an increased risk for chronic diseases and mortality. Some personality characteristics seem to increase risk for negative health outcomes and worse prognoses during disease course (i.e., Powers and Oltmanns, 2012; Wimmelmann et al., 2018). Recently, due to the worldwide increase of the prevalence of overweight and obesity and their all-cause mortality (Ezzati et al., 2018), particular attention has been paid to the link between overweight/obesity and personality features.

Obesity is the result of complex interactions between genetics and environmental and psychological factors (Sutin et al., 2011). Several aspects of psychological functioning, like depressive symptoms, anxiety, and its related somatic manifestations, are involved in body mass index (BMI) and weight gain (Luppino et al., 2010; Pazzagli et al., 2013; Tambelli et al., 2017).

Alongside the significant role of these psychopathological dimensions, in the comprehension of the psychological functioning of persons with overweight/obesity, several studies considered that interindividual differences in overweight/obesity susceptibility depends also on personality features and assessed their predictive role in overweight/obesity (i.e., Heaven et al., 2001; Terracciano et al., 2009; Gerlach et al., 2015; Doering, 2019). Therefore, in addition to the abovementioned factors, weight could be influenced also by personality features, that is, the individuals’ patterns of behavior, thinking, and feelings (Kazdin, 2000).

In order to investigate personality–BMI associations, until now, studies have mainly focused on broad personality traits. In particular, the Big Five Personality Trait model of personality structure has been used, which includes five broad traits: neuroticism, extraversion, openness, agreeableness, and conscientiousness. Obesity has been found to be associated with some personality traits but findings are inconsistent (Gerlach et al., 2015; Sutin and Terracciano, 2016a, 2019; Wimmelmann et al., 2018; Bagnjuk et al., 2019; Vainik et al., 2019). To date, several cross-sectional studies have been conducted showing mixed results: if conscientiousness tends to be associated with healthier BMI and high neuroticism tends to be related to a higher BMI and risk of obesity, the association between the other personality traits and weight is less clear (Sutin et al., 2011; Jokela et al., 2013; Sutin and Terracciano, 2016b, 2019). Up to now, studies showed that some of this variability could arise from moderators of BMI–personality associations.

Findings showed gender-related differences in the associations between BMI and the broad personality traits (Faith et al., 2001; Provencher et al., 2008; Soto et al., 2011; Vainik et al., 2019). Even if with a very small magnitude of the associations, Faith et al. (2001) showed that increasing BMI was significantly associated with more neuroticism and less extraversion among women, and with more extraversion and psychoticism among men. In line with previous studies, in the present study, gender has been tested as an independent variable.

Moreover, it has been suggested that the inconsistent association between obesity and broad personality domains could also be due to the possibility that the links pertain only to some facets of these domains. Specifically, in a recent meta-analysis, Vainik et al. (2019) found that BMI was associated with 15 specific facets of the main personality domains. However, these associations were small, except for impulsiveness, which had the strongest association with BMI. Overall, data showed that these specific aspect-based personality “risk” scores were a powerful predictor of BMI than the broader personality domains. These findings showed the need for further studies focused on the link between BMI and a wider set of personality characteristics than the broad five domains alone. This can be addressed by using a multidimensional measure of personality and psychopathology such as Personality Assessment Inventory (PAI; Morey, 1991). The use of a measure of personality and psychopathological dimensions, which has also been shown to describe the covariance in normal personality traits and personality disorders, could be of considerable importance to deepen the association between personality and obesity. Furthermore, the PAI, assessing personality on a multidimensional level through the clinical scales, may be particularly useful in facing the complex psychological functioning of people with high BMI and to plan targeted interventions taking into account this complexity.

Given the worldwide increase of obesity, and its subsequent health conditions, it could be helpful to broaden the current knowledge concerning personality features in individuals with obesity in order to enhance the comprehension of trajectories toward unhealthy lifestyles and to develop tailored interventions (Gerlach et al., 2015; Wimmelmann et al., 2018). To our knowledge, this is the first study aiming to investigate the associations between overweight/obesity – operationalized as BMI and personality and psychopathological dimensions through the clinical scales of PAI comparing a sample of outpatients with overweight/obesity with a matched non-clinical sample.

The aim was addressed in two ways. First, we investigated the main differences between the two samples in the personality and psychopathological dimensions assessed by the PAI clinical scales and subscales. Secondly, we evaluated which specific personality and psychopathological dimensions were the better predictors of overweight/obesity risk. As previous studies found gender-related differences in personality traits, the role of gender was taken into account in this study. Based on the aforementioned meta analytic study (Sutin et al., 2011; Vainik et al., 2019), showing impulsiveness having the strongest association with BMI, it was hypothesized that borderline features as measured by the PAI were associated with BMI, with impulsivity being one core aspect of such features. In line with previous studies mentioned above, it was furthermore hypothesized that individuals with overweight/obesity would show higher depressive symptoms, anxiety, and its related somatic manifestations in comparison with the matched non-clinical sample.

## Materials and Methods

### Participants and Procedures

**FIGURE 1:**
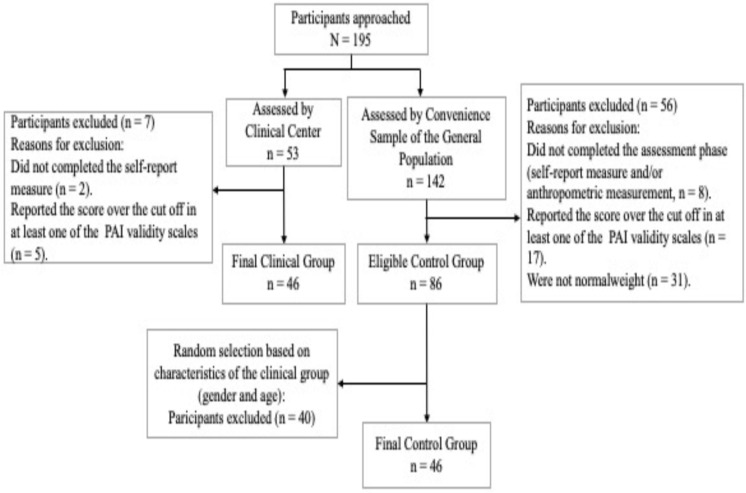
Flow diagram of enrolled participants.

The total sample (Figure 1) consisted of 92 Caucasian subjects, 28 males and 64 females, and the mean age was 55.14 (SD = 12.45; Min = 21 and Max = 76). These subjects are composed of two groups matched by gender (14 males and 32 females each group) and age (*t* = −0.139; *p* = 0.889). The clinical group consisted of 46 outpatients (mean age = 55.83; SD = 12.84) with overweight or obesity recruited at C.U.R.I.A.Mo the Healthy Lifestyle Institute for adulthood overweight–obesity treatment of the University of Perugia in Italy. The inclusion criteria to gain access at C.U.R.I.A.Mo was having a BMI ≥ 25 (mean = 36.08; SD = 7.14). The control group consisted of 46 subjects (mean age = 54.96; SD = 12.60), randomly selected from a convenience sample of general population with normal weight; the inclusion criteria to take part in this group was a BMI < 25 (mean = 21.88; SD = 1.73).

To ensure the accuracy of the self-report information about their psychological status, only subjects who did not report any score over the cutoff in each PAI validity scale (cutoff: Inconsistency = 72; Infrequency = 74; Negative Impression = 91; Positive Impression = 62) were included. All participants, having signed the informed consent/assent after being briefed on the study according to the Ethical Principles of Psychologists and Code of Conduct of the American Psychological Association (2010), completed some Italian versions of self-report measures to assess their psychological functioning. For the clinical group, the measures were included within the assessment phase required from the Center; for the control group, subjects were asked to participate in the study by asking them to accept to be measured for weight and height and to fill out questionnaires. The anthropometric measures were acquired in both groups by trained investigators. No incentive reward was given for the participation. The study was approved by the local Ethics Committee (CEAS Umbria Region, HREC number 1/10/1633).

### Measures

#### Anthropometric Measures

Participant’s height and body weight were assessed by physicians using standard techniques (Habicht, 1974) to calculate BMI [weight in kg/(height in m × height in m)].

#### Self-Report Measures

**FIGURE 2:**
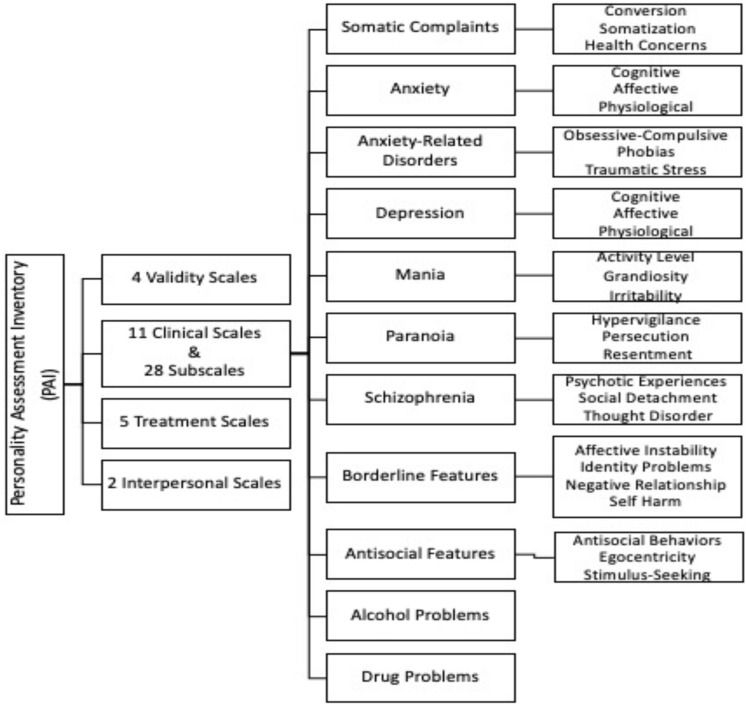
PAI structure.

Personality Assessment Inventory (Morey, 1991): It is a self-report consisting of 344 four-point Likert type items (0–3) forming 22 different scales. Figure 2 shows the conceptualizing of the PAI structure. In this study, 4 validity scales (Inconsistency, Infrequency, Negative Impression, and Positive Impression) and 11 clinical scales and related subscales were analyzed. Each of the main clinical scales, except for Alcohol Problems and Drug Problems, is made up of three or four subscales that assess the specific symptoms and features with an equal number of items (8).

The raw scores for all scales and subscales are obtained by adding the scores of each individual item and subsequently they are converted in the form of linear T scores that have a mean score of 50T and a standard deviation of 10T. Higher scores reflect greater difficulties in the personality and psychopathological dimensions.

The PAI has demonstrated good internal consistency (Cronbach’s α median value of 0.81) and test–retest reliability (mean value of 0.82) (Morey, 1991). In this study, the Italian version of PAI was administered, showing adequate internal consistency (Cronbach’s α mean value for all scales is 0.61, while it is 0.70 for clinical scales only) and test–retest reliability (mean value is 0.74 for all scales and 0.76 for clinical scales only) (Zennaro et al., 2015). Previous research has shown that the PAI maintains adequate psychometric properties in clinical samples as well (Siefert et al., 2009).

### Data Analysis

As in previous studies (Terracciano et al., 2009; Siu-Man and Xuebing, 2017), different analyses were performed in order to meet the aim of the present study.

Due to the non-normality distribution of 19 out of 22 of the analyzed variables, non-parametric statistics were performed. The only three clinical scales that show normal distribution were Mania, Borderline Features, and Antisocial Features.

In a preliminary step, a series of the analysis of variance of aligned rank transformed (ART) were run to identify the differences on the PAI clinical scales, with group (control vs. clinical) and gender (male vs. female) as independent variables. In order to examine more in depth the differences that arose, the ART for each subscale of the significant different clinical scales was performed, with group (control vs. clinical) and gender (male vs. female) as independent variables also in this case. Moreover, the interaction between the independent variables was tested.

Effect size was measured using partial eta-squared, in which small, medium, and large effect size were 0.01, 0.06, and 0.14, respectively (Cohen, 1988). According to previous studies (Pignolo et al., 2018), only results showing a medium and high effect size were considered and discussed.

Finally, to investigate the predictor role of personality and psychopathological dimensions on overweight and obesity, logistic regression analysis was used on the whole sample. More specifically, in the first step, three separate logistic regressions, with clinical scales emerging as significant from the ART analysis, were performed. Subsequently, three separate logistic regressions with the subscales of the abovementioned scales were run. Findings were reported as odds ratios (ORs) with 95% CI. Statistical significance requires a *p* value < 0.05. RStudio (Version 1.0.143) and Statistical Package for Social Science (IBM SPSS Version 21, SPSS Inc., 2009) were used for data analysis.

## Results

### Analysis of Variance of Aligned Rank Transformed

Aligned rank transformed (Table 1) revealed significant differences in some PAI’s clinical scales for group and for gender.

**TABLE 1:** Analysis of variance of aligned rank transformed: means and standard deviations of PAI clinical scales and subscales separately for gender and group. *F*, *p* value, and effect size (η_*p*_^2^).

	Males		Female		Gender Males vs. Females			Clinical group		Control group		Group clinical vs. control			Gender × group			Cronbach’s α
PAI clinical scale	Mean	SD	Mean	SD	*F*_(1,88)_	*p*	η_*p*_^2^	Mean	SD	Mean	SD	*F*_(1,88)_	*p*	η_*p*_^2^	*F*_(1,88)_	*p*	η_*p*_^2^	α
Somatic complains	52.72	6.93	54.09	10.16	0.024	0.877	0.004	57.09	9.88	50.24	7.23	14.23	0.001	0.122	0.062	0.803	0.000	0.80
Anxiety	51.79	9.21	58.89	11.18	11.72	0.001	0.089	57.85	11.92	55.46	10.11	0.701	0.404	0.015	0.330	0.567	0.004	0.86
Anxiety-related disorders	47.24	8.58	55.67	12.40	11.69	0.001	0.109	54.30	12.44	51.72	11.43	0.939	0.335	0.007	0.312	0.578	0.002	0.70
Depression	51.90	7.40	58.22	11.12	6.85	0.010	0.084	59.30	11.30	53.15	8.66	6.88	0.010	0.069	0.323	0.572	0.004	0.85
Mania	55.27	13.62	51.98	11.13	1.40	0.239	0.017	52.39	12.31	53.65	11.77	0.325	0.567	0.000	1.57	0.213	0.017	0.77
Paranoia	57.07	11.30	54.90	9.83	0.539	0.464	0.011	57.50	11.89	53.67	8.11	1.64	0.204	0.032	0.017	0.895	0.000	0.74
Schizophrenia	50.07	8.95	50.51	9.95	0.024	0.877	0.000	51.93	10.50	48.80	8.44	1.56	0.214	0.016	0.418	0.519	0.006	0.73
Borderline features	49.69	7.32	51.76	9.18	0.607	0.437	0.012	53.93	8.88	48.28	7.49	8.49	0.004	0.095	0.023	0.878	0.000	0.78
Antisocial features	51.65	7.81	48.25	7.16	5.08	0.027	0.046	49.48	6.34	49.17	8.57	0.332	0.565	0.003	0.729	0.192	0.008	0.64
Alcohol problems	46.00	4.56	47.00	4.46	1.44	0.233	0.011	46.28	3.80	47.09	5.10	0.013	0.910	0.002	1.50	0.222	0.014	0.53
Drug problems	49.86	6.93	50.73	8.53	0.047	0.828	0.002	51.48	8.81	49.43	7.13	0.224	0.639	0.008	0.347	0.557	0.006	0.55
**PAI clinical subscale**																		
Somatic complains subscales																		
SOM-conversion	52.52	7.57	54.32	11.99	0.001	0.980	0.006	56.67	11.82	50.83	8.83	8.01	0.005	0.066	0.006	0.936	0.000	0.65
SOM-somatization	53.45	9.49	55.19	9.89	0.518	0.473	0.007	57.61	10.55	51.68	7.83	6.46	0.013	0.078	0.009	0.926	0.000	0.53
SOM-health concerns	51.10	8.98	51.43	10.16	0.118	0.731	0.000	54.39	10.36	48.24	8.10	10.69	0.001	0.092	0.303	0.583	0.004	0.71
Depression subscales																		
DEP-cognitive	51.72	9.77	55.30	10.95	2.68	0.105	0.024	56.61	12.27	51.74	8.24	3.00	0.086	0.042	0.437	0.510	0.004	0.65
DEP-affective	53.07	9.36	57.54	12.78	2.79	0.098	0.031	58.98	13.25	53.28	9.81	4.44	0.038	0.041	0.356	0.552	0.004	0.77
DEP-physiological	50.03	5.53	57.76	9.44	19.56	0.000	0.165	57.87	9.39	52.78	8.17	7.59	0.007	0.068	0.605	0.552	0.004	0.69
Borderline features subscales																		
BOR-affective instability	47.38	6.68	52.67	9.97	8.07	0.005	0.070	53.09	10.08	48.91	8.17	4.62	0.034	0.045	0.007	0.932	0.000	0.63
BOR-identity problems	50.83	7.40	53.62	10.10	1.45	0.231	0.017	55.50	10.88	49.99	8.55	6.14	0.015	0.056	0.185	0.668	0.003	0.60
BOR-negative relationship	53.69	10.10	53.90	9.87	0.123	0.727	0.000	57.13	10.68	50.54	7.83	6.16	0.015	0.100	0.060	0.807	0.000	0.65
BOR-self-harm	46.31	8.93	43.62	7.42	2.65	0.106	0.026	45.11	8.10	43.83	7.89	2.46	0.120	0.013	2.82	0.096	0.009	0.53

*Cronbach’s α of all measures analyzed.*

**p* < 0.05 significant difference.*

*η_*p*_^2^ ≥ 0.01 small effect size; ≥0.06 medium effect size; ≥0.14 large effect size.*

*SOM, Somatic Complaints; DEP, Depression; BOR, Borderline Features.*

With respect to group category, subjects with overweight and obesity showed higher scores in the Somatic Complaints, Depression, and Borderline Features scales than the control group’s subjects with medium to large effect size. Regarding differences between gender, there were statistically significant differences in the mean scores in four scales but only three with medium to large effect size: females scored higher on Anxiety, Anxiety-Related Disorder, and Depression than males.

More in depth, ART revealed the effect of group and gender in some of the clinical subscales analyzed.

More specifically (Table 1), the clinical group reported higher scores in all subscales that assess the characterizing symptoms of the Somatic Complaints scales: Conversion, Somatization, and Health Concerns, with medium to large effect size.

With regard to the characterizing symptoms of the Depression clinical scales, the subjects with overweight or obesity showed higher presence of physiological symptoms than the control group, with medium effect size.

Lastly, with regard to Borderline Features, the clinical group reported higher scores in three specific subscales but only the Negative Relationship subscale with medium effect size.

In regard to the differences between gender, there was a statistically significant difference in two subscales, both with medium to large effect size (DEP-Physiological and BOR-Affective Instability). In particular, females reported higher scores in Physiological (a Depression subscale) and in Affective Instability (a Borderline Features subscale).

### Logistic Regression

Table 2 shows logistical regression analysis for the clinical scales highlighted as different between overweight/obesity patients and non-clinical subjects by ART, and all their subscales were entered as predictors of overweight and obesity. Since no interactions between group and gender were shown, the latter was not included in the regression models. The results show that all models were significant.

**TABLE 2:** Logistic regression models of PAI clinical scales and subscales in control vs. clinical group.

Clinical scales	β	χ^2^	Wald statistics	OR	95% CI
Model 1		14.09***			
Somatic complains	0.10		10.84**	1.10	1.04–1.17
Model 2		6.35**			
Depression	0.06		7.27*	1.06	1.02–1.11
Model 3		10.51**			
Borderline features	0.09		8.88**	1.09	1.03–1.15

**Clinical subscales**	**β**	**χ^2^**	**Wald statistics**	**OR**	**95% CI**

Model 1		14.29**			
SOM-conversion	0.021		0.572	1.02	0.968–1.08
SOM-somatization	0.044		2.32	1.04	0.988–1.10
SOM-health concerns	0.050		2.93	1.05	0.993–1.11
Model 2		8.91			
DEP-cognitive	0.019		0.375	1.02	0.960–1.08
DEP-affective	0.012		0.166	1.01	0.956–1.07
DEP-physiological	0.048		2.71	1.05	0.991–1.11
Model 3		13.95**			
BOR-affective instability	0.015		0.231	1.01	0.954–1.08
BOR-identity problems	0.034		1.32	1.03	0.976–1.10
BOR-negative relationship	0.065		5.70*	1.07	1.01–1.12
BOR-self-harm	−0.014		0.217	0.986	0.928–1.05

*Effect was statistically significant at *p* < 0.05.

**Effect was statistically significant at *p* < 0.01.

***Effect was statistically significant at *p* < 0.001.

*SOM, Somatic Complains; DEP, Depression; BOR, Borderline Features.*

Data reported in Table 2 show that all clinical scales included (Somatic Complaints, Depression, and Borderline Features) contribute significantly to the increased risk of belonging to the clinical group. In particular, an OR of 1.10 indicated a significant increase (95% CI = 1.04–1.17; β = 0.10; *p* < 0.01) in the odds of being in the clinical group for each unit increment of Somatic Complaints. A similar significant increase in the odds of being in the clinical group was found for each unit increment of Depression (OR = 1.06; 95% CI = 1.02–1.11; β = 0.06; *p* < 0.05) and for each unit increment of Borderline Features (OR = 1.09; 95% CI = 1.03–1.15; β = 0.09; *p* < 0.01).

To investigate more in detail which personality and psychopathological dimensions played a key role in overweight and obesity, all the subscales of the clinical scales previously considered have been included as predictors.

Results highlighted that the only subscale that increased the risk of overweight and obesity was the Negative Relationship belonging to the Borderline Features (OR = 1.07; 95% CI = 1.00–1.12; β = 0.06; *p* < 0.01); no other subscales of this specific scale (Affective Instability, Identity Problems, and Self-harm) were statistically significant.

Furthermore, no subscale characterizing the Somatic Complaints (Conversion, Somatization, and Health Concerns), as well as the Depression subscales (Cognitive, Affective, and Physiological) considered individually, was statistically significant.

## Discussion

The main aim of the current study was to investigate if specific personality and psychopathological dimensions assessed by the PAI were associated with overweight and obesity. This tool allows us to assess personality conceived as a breadth construct (Morey, 1991, 2007), allowing the knowledge of its complexity on a multidimensional level.

In regard to the differences between the two samples in the personality and psychopathological dimensions assessed by the PAI clinical scales and subscales, findings showed significant differences with higher scores in subjects with overweight/obesity compared with a matched non-clinical group for Somatic Complaints, Depression, and Borderline Features with some statistically significant differences between gender.

Consistent with the hypothesis and in line with studies indicating the common presence of somatic symptoms and obesity in primary care settings and their strong association, our findings showed higher somatic symptoms, conversion, somatization, and health concerns in the clinical group.

Differently from what was hypothesized, no significant differences emerged between the two groups with regard to anxiety scale. As the PAI’s somatic complaints scale is composed of conversion, somatization, and health concerns, this result might be understood in the light of data showing the significative role played by health concerns, rather than anxiety sensitivity, in persons with obesity (Fergus et al., 2018). The present study seemed to support the relevance of health anxiety in the increased somatic symptoms among patients with obesity. Future studies with larger samples should confirm this preliminary data.

The association between overweight/obesity and depression has been repeatedly established in studies. Obesity was found to increase the risk of depression, and depression was found to be predictive of developing overweight or obesity (Luppino et al., 2010). The issue of causality remains unclear, and data seemed to indicate a bidirectional causal pathway between overweight, obesity, and depression (Faith et al., 2011; Pazzagli et al., 2013). When leading treatment interventions, in order to reduce these specific health conditions, Somatic Complaints and Depression, which are factors that respond well to treatment, must be taken into account.

In regard to Borderline Features, studies have demonstrated an association between some personality traits and obesity as well as their prognostic influence on weight course (Gerlach et al., 2016). Recent papers showed that borderline pathology is an important risk factor for serious health problems in later adulthood, including obesity, a condition that is linked to many chronic health diseases (Ezzati et al., 2018; Doering, 2019). Powers and Oltmanns (2013), with an epidemiologically based sample, found that borderline features were significantly related to reported presence of heart disease, arthritis, and obesity. Furthermore, they found that BMI fully mediated the relation between Borderline Features and arthritis. Specifically, authors considered obesity as one pathway that leads to more health problems among individuals with borderline symptoms. A possible data explanation is that difficulties with emotion regulation and impulsivity could have a strong effect on the development of obesity and thus might contribute to health problems. Emotion dysregulation has been hypothesized to play a central role in the etiology and development of borderline functioning (Glenn and Klonsky, 2009; Stepp et al., 2014). Furthermore, the issue of emotion dysregulation has recently received increasing support in studies on obesity and on its association with interpersonal problems (e.g., Frankel et al., 2012; Herr et al., 2013; Hughes et al., 2015; Aparicio et al., 2016; Escandón-Nagel et al., 2018).

Regarding differences between gender, according to previous studies, the present data showed differences for gender in some psychopathologies and personality features. The females referred higher general distress and affective disorder than males (Leach et al., 2008; Faravelli et al., 2010; Pignolo et al., 2018), particularly higher levels of anxiety, anxiety-related disorders, and depression. Moreover, in both clinical and non-clinical subjects, females showed higher level of borderline affective instability (also called emotional lability) than males. These data are in line with earlier studies conducted in clinical populations (Gunderson and Links, 2007) but in contrast with most of the studies on community sample that did not highlight differences between males and females (Paris, 2010). Due to the lack of interaction between gender and clinical/control group, gender has not been included in the subsequent analyses.

The present findings with logistic regression specifically showed that the PAI subscale Negative Relationship of the Borderline Features contributed to the increased risk of belonging to the group with overweight and obesity. The results highlighted the importance of taking into account personality functioning particularly in the assessment of treatment-seeking patients with overweight/obesity.

Morey (1991) described subjects with high scores in the Negative Relationship subscale of Borderline Features as individuals with a history of intense and ambivalent relationships, showing several difficulties in attachment relationships. Such individuals, in fact, often feel that others do not recognize their needs with subsequent feelings not only of disappointment but also of being betrayed and exploited. Frequently, these subjects look at current and future relationships with mistrust and fear of being abandoned or rejected. These specific characteristics are consistent with studies that place obesity within the framework of attachment theory.

These assumptions are in line with results of a recent meta-analytic review on the significance of attachment quality in obesity highlighting that BMI is negatively associated with attachment security (Diener et al., 2016). A possible explanation for the impact of attachment quality on overweight and obesity risk is considered the underdevelopment of emotion regulation (Mazzeschi et al., 2014; Nancarrow et al., 2018).

Consistent with the present results on the quality of interpersonal relationships, studies showed that individuals with obesity reported elevated interpersonal distress (e.g., Lo Coco et al., 2012). Some studies reported the presence of maladaptive schemata related to social isolation, shame, and failure to achieve and an association between overeating and early maladaptive schemas (Anderson et al., 2006; Da Luz et al., 2017; Imperatori et al., 2017). Specifically, a recent study showed that individuals with overweight and obesity reported more intense abandonment, dependence, subjugation, and insufficient self-control schemas, compared with normal-weight subjects (Basile et al., 2019).

The present findings suggest that overweight and obesity are rooted within patients’ personality features. The study of personality–overweight/obesity link showed that PAI’s subscale Negative Relationship of the Borderline Features scale was the strongest predictor of BMI. As reported, emotion dysregulation is considered to play a central role both in borderline functioning and in individuals with overweight or obesity. Hence, the assessment of personality through a dimensional measure and the findings on the personality–BMI associations could provide starting points for tailoring interventions for overweight and obesity. Specifically, individuals with obesity seem to have the tendency to establish extremely dependent and enmeshed relations (Bruch, 1975), characterized by feelings that their needs are not recognized and by mistrust in future relations within a wider interpersonal and emotional instability, typical of borderline functioning (Lingiardi and McWilliams, 2017), hence the importance to carefully assess patients’ personality and interpersonal functioning in the treatment of adults with obesity for long-term success of weight loss and health promotion interventions.

A deeper understanding of these personality features might be important to be taken into account considering that a person with borderline features shows poor adherence to psychological and medical treatment recommendations (Powers and Oltmanns, 2013) that can complicate the course of several diseases due to the negative perception of health and poor health-related behavior and lifestyle (El-Gabalawy et al., 2010).

The present results must be interpreted in light of some methodological limitations. First, the study is exploratory in nature and the findings need to be replicated with a larger sample before firm conclusions can be drawn. The clinical sample consisted of treatment-seeking individuals with overweight or obesity. Studies suggested that a treatment-seeking sample with overweight/obesity may differ from subjects with overweight/obesity in the community with regard to personality functioning such as illness awareness, illness behavior, and treatment rejection (e.g., Wadden et al., 2002). Consequently, results are not generalizable to individuals with overweight or obesity not seeking treatment. Furthermore, as low reliability was observed in two subscales (SOM-Somatization and BOR-Identity Problems) taken into account in the Logistic Regression models, the present findings should be interpreted with caution.

Moreover, in regard to the direction of causality, with the present study being a cross-sectional study, data are limited to concurrent BMI (not to future BMI). Therefore, the associations highlighted here do not offer evidence on the directionality of the relationships. In future research, longitudinal data will more definitely predict associations between the selected variables. Finally, the study aimed to focus on the associations between overweight/obesity and the clinical scales of the PAI, but many other factors might influence individuals with overweight or obesity (e.g., demographic factors as socioeconomic status). To date, studies demonstrated an association between personality traits and overweight/obesity as well as their prognostic influence on weight course. To our knowledge, this is the first study that aims to explore the contribution of specific personality features assessed with a dimensional measure of personality and psychopathology to the prediction of subjects’ weight.

The present study may have important clinical implications. It highlighted the importance of focusing on the specific characteristics of interpersonal relationship in the treatment since patients’ prevalent interpersonal models could affect the relationship with the clinician. Also, the importance of considering emotional instability typical of borderline functioning for long-term success interventions should be considered. Overall, the results may provide additional evidence for the importance of assessing personality functioning with a dimensional measure of personality and psychopathology in order to allow treatment to rapidly focus on patterns that need to change.

## Data Availability Statement

The datasets generated for this study are available on request to the corresponding author.

## Ethics Statement

The studies involving human participants were reviewed and approved by the local Ethics Committee (CEAS Umbria Region, HREC number 1/10/1633). The patients/participants provided their written informed consent to participate in this study.

## Author Contributions

LB: made contributions to data collection, literature review, data analysis, data interpretation, and co-wrote article. CP: made contributions to the conception and design of the work, data collection, literature review, revised data interpretation, wrote substantive section of article, and addressed reviewer comments. ED: made contributions to the conception and design of the work, and data collection. GC: participated in the revision of the manuscript. AG: participated in data analysis and commented on previous draft of the manuscript. CM: made substantial contributions to the conception and design of the work, data collection, revised it critically for important intellectual content, and approved the final version to be published. All authors contributed to the article and approved the submitted version.

## Conflict of Interest

The authors declare that the research was conducted in the absence of any commercial or financial relationships that could be construed as a potential conflict of interest.
